# Arthroscopic Lateral Meniscus Posterior Root Repair Using All‐Suture Anchor and Modified Transtibial Pull‐Out Technique

**DOI:** 10.1002/atn2.70111

**Published:** 2026-06-28

**Authors:** Chen‐Jian Peng, Shu‐ya Sun, Peng Xue, Jian‐Ning Zhao, Shuo Chen

**Affiliations:** ^1^ Nanjing Hospital of Chinese Medicine Nanjing Sports Hospital Nanjing Hospital of Chinese Medicine Affiliated to Nanjing University of Chinese Medicine Nanjing China

## Abstract

Lateral meniscus posterior root tears lead to disruption of hoop tension, elevation of contact pressure, increased knee instability, and accelerated cartilage degeneration of articular cartilage. Anatomical repair is required to re‐establish normal knee biomechanics and to slow the progression of osteoarthritis. A modified transtibial pull‐out technique using an all‐suture anchor is described. The tail suture is applied for root fixation, creating a suture ball on the meniscal surface, while a 2 mm tibial tunnel is created at the anatomic footprint to achieve refixation. This technique provides stable, minimally invasive, and anatomical repair, minimizing bone loss, avoiding tunnel interference with anterior cruciate ligament grafts, and promoting biological healing.

## Graphical abstract

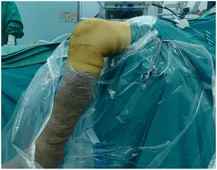

**VIDEO 1:** This is a patient with a lateral meniscus posterior root tear in the right knee. Initially, the tear is diagnosed as Laprade type II‐2C by arthroscopic exploration. The surrounding tissues are cleared to facilitate easy repositioning of the meniscal root. A point‐to‐point transtibial drill guide is used to create a 2.0‐mm tibial tunnel in the anatomical footprint of the lateral meniscus posterior root. A spinal needle is used to pass the PDS suture through the tibial tunnel and into the knee. A suture hook loaded with PDS penetrates the meniscus at two points of the lateral meniscus posterior root from superior to inferior in a vertical direction, with a 3‐5‐mm interval between them. Then, the two limbs of the all‐suture anchor are passed into the meniscus by PDS, and the suture ball is positioned on the superior aspect of the LMPR. The two anchor sutures are tied to the carrier suture (the first transtibial PDS) and pulled through the tibial tunnel, then fixed using an Endobutton. Finally, a probe is used to confirm the stable attachment of the LMPR under direct visualization. Video content can be viewed at https://doi.org/10.1002/atn2.70111.

Meniscal posterior root tears, a specific type of meniscal injury, account for about 10% to 21% of all meniscal tears and have garnered significant attention as a focus of sports medicine in recent years. Meniscal root tears are defined as radial tears within 1 cm of the root attachment site or complete avulsion of the root from bone or soft tissue. Biomechanical investigations have established the critical role of the meniscus in the knee, especially in transforming axial tibiofemoral loads into hoop stresses. This process depends on the structural integrity of the meniscal root.^1^ It has been shown that posterior root tears create biomechanical conditions comparable to those observed after meniscectomy, thereby accelerating the development of knee osteoarthritis; in addition, posterior root tears of the lateral meniscus have been reported to worsen anterior knee instability.^2^ Therefore, restoring the structural integrity of the lateral meniscus posterior root is crucial for preserving knee function.

Previous studies have reported that surgical techniques for repairing meniscal posterior root tears include the suture anchor repair technique and the transtibial pull‐out technique.^2^, ^3^, ^4^ Although suture anchor techniques are effective, they are technically challenging, require an additional posterior approach, and carry a higher risk of neurovascular injury.^3^ The traditional transtibial pull‐out technique, although simpler in nature, requires a 4.5 mm tibial tunnel. The 4.5 mm tunnel allows greater suture capacity but also increases bone mass loss and may affect the graft when combined with anterior cruciate ligament injury.^4^

In this technique, we describe a modified transtibial pull‐out approach using an all‐suture anchor to facilitate repair of meniscal posterior root tears, with the suture ball placed on the surface of the meniscus (Video 1). A 2.0 mm tibial tunnel is created within the anatomical footprint area, and the suture is subsequently pulled out for external fixation, thereby achieving anatomical repair of the posterior root of the lateral meniscus.

## SURGICAL TECHNIQUE

### Patient Positioning and Portal Preparation

**FIGURE 1:**
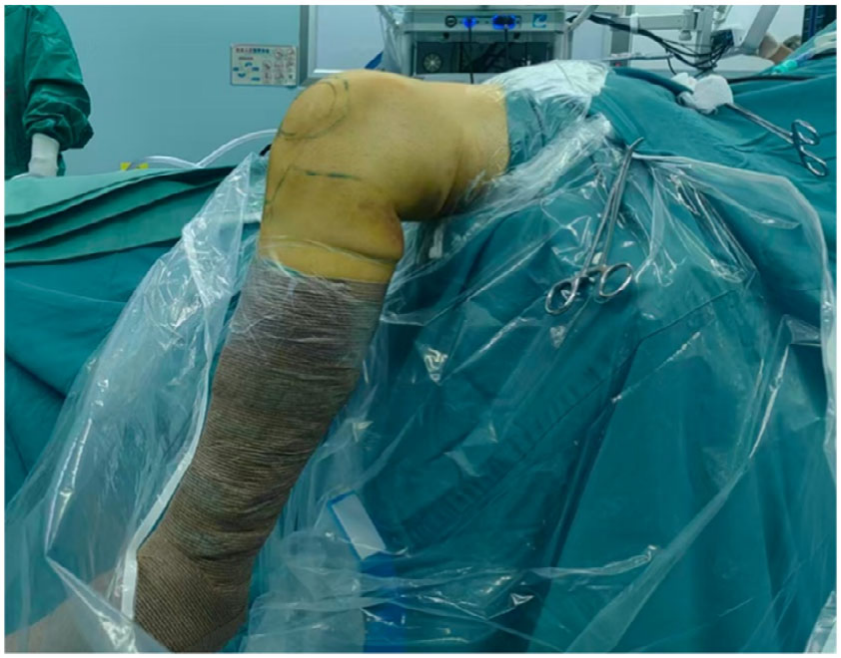
Patient positioning—operative leg suspended on a leg holder (left knee).

The patient is positioned supine under spinal anesthesia, with the operative limb suspended on a leg holder. A tourniquet is placed around the thigh, and the surgical field is prepared under sterile conditions. The operative limb is prepped and draped in the standard manner (Figure 1). Standard anterolateral and anteromedial portals are then created for arthroscopic evaluation.

### Diagnostic Arthroscopy and Preparation of the Meniscus

**FIGURE 2:**
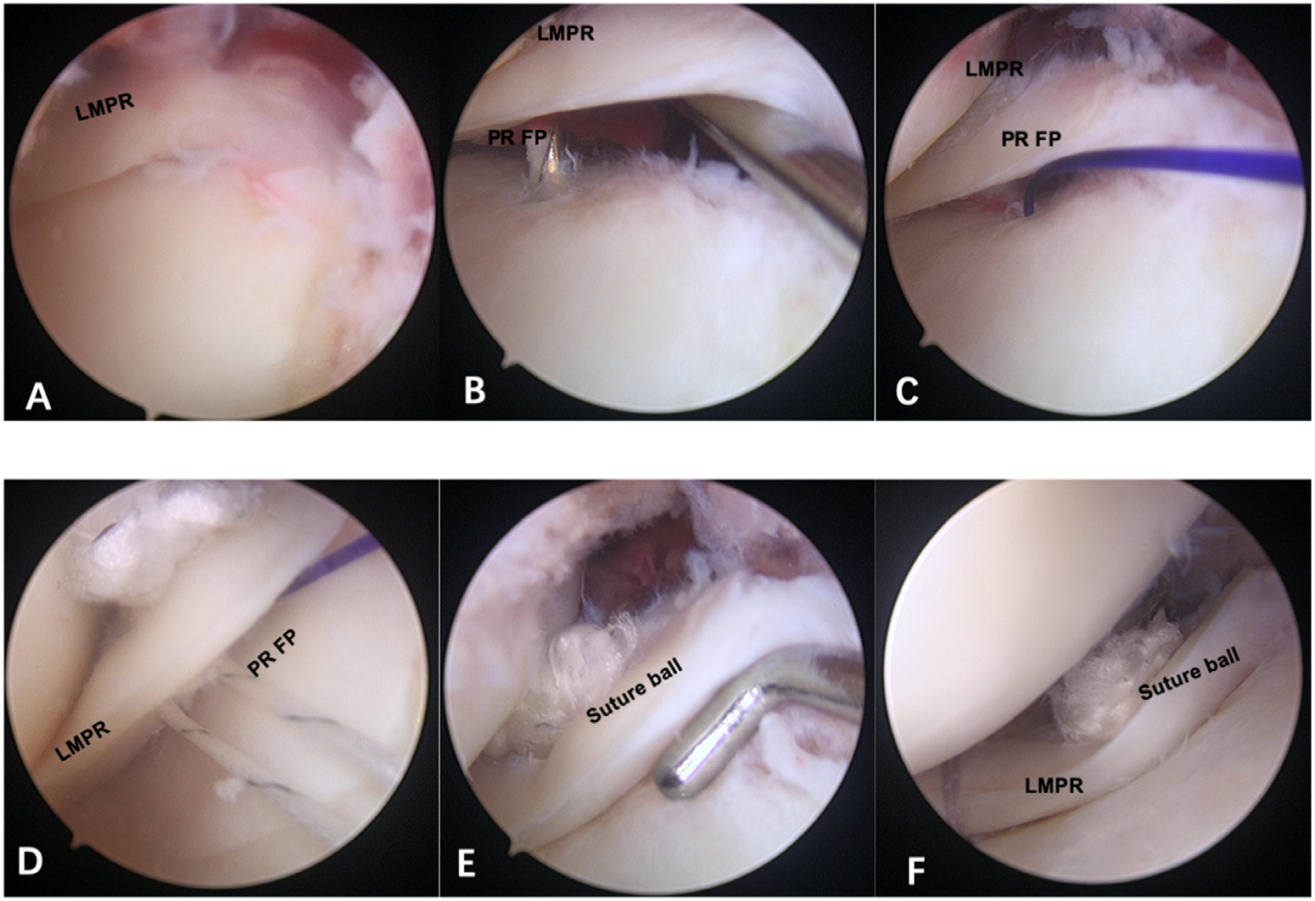
LMPRT arthroscopic view (right knee). (A) Diagnostic arthroscopy and preparation of the meniscus. (B) Bone bed preparation and tibial tunnel drilling in the footprint area. (C) Pass the PDS through the tibial tunnel. (D) All‐suture anchors are passed into the meniscus. (E) Suture ball is positioned on the superior of the LMPR. (F) LMPR is repaired and refixed in anatomic position. (LMPR, lateral meniscus posterior root; LMPRT, lateral meniscus posterior root tear; PDS, polydioxanone suture; PR FP, posterior root footprint.)

Under arthroscopic observation using a 30° scope (CONMED, Tuttlingen, Germany), a probe is used to assess the attachment of the meniscal root, and a shaver (CONMED, Largo, FL) is employed to clear surrounding tissues to facilitate easy repositioning of the meniscal root. The bone bed in the footprint area is pretreated with a curette or grinding head to promote healing (Figure 2A).

### Additional Instruments

**FIGURE 3:**
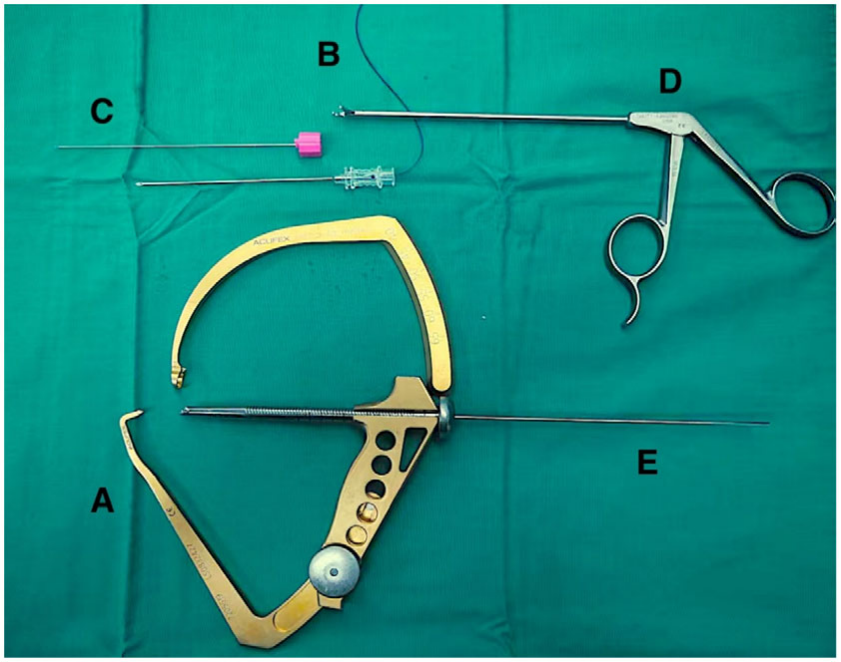
Additional instruments. (A) Point‐to‐point or point‐to‐loop drill guide. (B) No. 0 PDS. (C) Spinal needle. (D) Suture passers. (E) 2.0 Kirschner wire. (PDS, polydioxanone suture.)

In addition to the basic arthroscopic setup, the following instruments are essential (Figure 3): a point‐to‐point or point‐to‐loop drill guide for transtibial drilling (Smith & Nephew, Mansfield, MA), no. 0 polydioxanone suture (PDS; ETHICON, Ciudad Juarez, Chihuahua, MX), a spinal needle, a Y‐Knot Flex 1.8 mm all‐suture anchor (CONMED, Largo, FL), an Endobutton (Smith & Nephew, Mansfield, MA), and a 2.0 Kirschner wire.

### Drilling Tibial Tunnel

The knee is positioned in a figure‐4 position, and the arthroscope is inserted through the anteromedial portal. A point‐to‐point drill guide for transtibial drilling is placed via the anterolateral portal, and a 2.0 mm tibial tunnel is created at the anatomical footprint of the lateral meniscus posterior root. A spinal needle is used to pass the PDS through the tibial tunnel and into the knee (Figure 2B,C).

### Meniscal Root Suturing and Fixation

A 1.8 mm Y‐Knot Flex all‐suture anchor is preformed into a suture ball. The arthroscope is inserted through the anteromedial portal, and a suture hook (Linvatec, Largo, FL) loaded with a no. 0 PDS is inserted through the anterolateral portal, and the tip of the hook penetrates the 2 points of the lateral meniscus posterior root from superior to inferior in a vertical direction, with a 3 to 5 mm interval between them. The 2 limbs of the all‐suture anchor are then passed into the meniscus using the PDS, and the suture ball is positioned on the superior surface of the lateral meniscus posterior root. The 2 anchor sutures are tied to the carrier suture (the first transtibial PDS), passed through the tibial tunnel, and fixed using an Endobutton. Finally, a probe is used to achieve stable attachment of the lateral meniscus posterior root under direct visualization (Figure 2D,E).

### Rehabilitation

Weight‐bearing: Partial weight‐bearing is allowed for the first 4 weeks, with gradual transition to full weight‐bearing after 6 weeks.

Range of Motion: The knee is immobilized in a hinged brace for 6 weeks. In the first 2 weeks, passive flexion is limited to 90°, reaching 120° by 6 weeks and full flexion by 8 weeks.

Strength Training: Muscle activation exercises begin on the first postoperative day. Three weeks after the operation, muscle strength training is conducted to exhaustion.

Coordination Training: Gluteal bridge exercises begin once the patient reaches 20% weight‐bearing capacity, progressing to balance exercises as tolerated.

## DISCUSSION

Lateral meniscus posterior root tears are highly prevalent among adolescent athletes and are frequently concomitant with anterior cruciate ligament injuries, which can be attributed to their unique anatomical characteristics.^1^, ^2^, ^5^, ^6^ The heightened mobility of the lateral meniscus, compared with the medial meniscus, allows its posterior root to transmit up to 85% of the load during knee flexion.^7^ Functionally, the lateral meniscus plays a role in load distribution, regulation of joint kinematics, reduction of contact stress, and protection of articular cartilage during both dynamic and static joint movements.^5^, ^6^, ^7^ Biomechanical evidence indicates that meniscectomy results in a marked increase in load transmission within the lateral compartment, making it more susceptible to accelerated degenerative changes than the medial compartment.^8^ Consequently, anatomical repair of the meniscal root is a vital treatment for restoring rotational stability and preventing osteoarthritis in the knee.^9^

Transtibial pull‐out techniques and suture anchor repair techniques are the most commonly used methods for meniscal posterior root repair, with both showing favorable clinical outcomes.^1^, ^2^, ^3^, ^4^ Transtibial pull‐out technique repair for lateral meniscus posterior root tears is associated with a low failure rate, as reflected by a revision rate of 6.7%.^10^ LaPrade et al.,^1^, ^4^, ^10^ in a 2.5‐year follow‐up study, revealed that the transtibial pull‐out technique effectively alleviated symptoms and significantly improved knee function, as evidenced by increased Lysholm and Tegner scores and a reduced Western Ontario and McMaster Universities Osteoarthritis Index score.

The conventional transtibial pull‐out method involves drilling a 4.5 mm tunnel from the proximal anterior tibia to the anatomical root attachment area. Sutures are passed through the torn meniscal posterior root of the meniscus, and retrieved through the tunnel, and secured over an Endobutton. Although this approach allows the release of biological factors from the tibial tunnel that may support healing, it also carries a risk of suture wear and reduced fixation stability. These limitations are mainly related to the bungee and wiper effects caused by the long and wide tunnel, as well as possible tunnel conflict during simultaneous ligament reconstruction.

In the modified traditional transtibial pull‐out approach, a 2 mm tibial tunnel is created and an all‐suture anchor is applied. Two suture limbs are passed through the torn meniscal posterior root, with the suture ball maintained on the meniscal surface. The sutures are then retrieved through the tunnel and tied down over an Endobutton. This modification provides several benefits: (1) restoration of the anatomical footprint with recovery of native contact mechanics; (2) 2‐point suture fixation of the posterior root, allowing a simple surgical procedure; (3) suture ball reinforcement to enlarge the contact area, resulting in strong fixation and reduced risk of avulsion; (4) release of growth factors induced by tunnel drilling, which improves the biological for healing; and (5) use of a small 2.0 mm diameter transtibial tunnel, limiting bone loss, preventing interference with concomitant ligament reconstruction, and reducing the “wiper effect.”

This technique integrates the advantages of 2 traditional methods, thereby simplifying and improving the anatomical repair of the lateral meniscus posterior root (Table 1). Despite these benefits, attention should be paid to the pitfalls and technical tips of this technique (Table 2), and the potential for a suture‐related “bungee” effect as well as the presence of a learning curve should be considered and addressed through future clinical studies for validation.

**TABLE 1:** Advantages and Disadvantages of Arthroscopic Lateral Meniscus Posterior Root Repair Using All‐Suture Anchor and Modified Transtibial Pull‐Out Technique

Advantages	Disadvantages
• Fixation of the meniscus allows restoration of the anatomical footprint	• Suturing at the posterior root carries a risk of cartilage injury
• Two‐point suture fixation of the posterior root provides procedural simplicity	• A learning curve exists, and suture‐related bungee effects may occur
• The suture ball enhances fixation strength and reduces the risk of avulsion	• A longer follow‐up period is necessary to clarify the safety and validity of this method
• A 2 mm tibial tunnel limits bone removal and decreases the wiper effect	

**TABLE 2:** Pitfalls and Tips of Arthroscopic Lateral Meniscus Posterior Root Repair Using All‐Suture Anchor and Modified Transtibial Pull‐Out Technique

Pearls	Tips
• A point‐to‐point or point‐to‐loop drill guide is used to ensure precise placement at the anatomical location	•Insufficient release of perimeniscal tissues may compromise accurate anatomical reduction of the posterior meniscal root
• Following creation of the tibial tunnel, bone debris at the drill exit is removed and the footprint region is refreshed, after which a spinal needle is introduced through the tibia	• Intra‐articular identification of the tibial tunnel using a spinal needle can be technically demanding
• Suturing of the posterior meniscal root is carried out using a suture hook or an appropriate suture‐passing device	•During arthroscopic procedures, confirmation should be made that the suture remains within the joint cavity
• A suture hook or suture passer is employed to complete the meniscal root suturing procedure	

## DISCLOSURES

The author (C‐J.P.) declares the following financial interests/personal relationships which may be considered as potential competing interests: C‐J.P. has a patent pending. The other authors (S.S., P.X., J‐N.Z., S.C.) declare that they have no known competing financial interests or personal relationships that could have appeared to influence the work reported in this article.
